# Fluvastatin synergistically enhances the antiproliferative effect of gemcitabine in human pancreatic cancer MIAPaCa-2 cells

**DOI:** 10.1038/sj.bjc.6602720

**Published:** 2005-07-19

**Authors:** G Bocci, A Fioravanti, P Orlandi, N Bernardini, P Collecchi, M Del Tacca, R Danesi

**Affiliations:** 1 Division of Pharmacology and Chemotherapy, University of Pisa, Via Roma, I-56126 Pisa, Italy; 2 Department of Human Morphology and Applied Biology, University of Pisa, Via Roma, I-56126 Pisa, Italy; 3 Division of Pathological Anatomy, Department of Oncology, Transplants and Advanced Technologies in Medicine, University of Pisa, Via Roma, I-56126 Pisa, Italy

**Keywords:** gemcitabine, fluvastatin, synergism, ras effectors, pancreatic cancer cells

## Abstract

The new combination between the nucleoside analogue gemcitabine and the cholesterol-lowering drug fluvastatin was investigated *in vitro* and *in vivo* on the human pancreatic tumour cell line MIAPaCa-2. The present study demonstrates that fluvastatin inhibits proliferation, induces apoptosis in pancreatic cancer cells harbouring a p21*ras* mutation at codon 12 and synergistically potentiates the cytotoxic effect of gemcitabine. The pharmacologic activities of fluvastatin are prevented by administration of mevalonic acid, suggesting that the shown inhibition of geranyl-geranylation and farnesylation of cellular proteins, including p21*rhoA* and p21*ras*, plays a major role in its anticancer effect. Fluvastatin treatment also indirectly inhibits the phosphorylation of p42ERK2/mitogen-activated protein kinase, the cellular effector of *ras* and other signal transduction peptides. Moreover, fluvastatin administration significantly increases the expression of the deoxycytidine kinase, the enzyme required for the activation of gemcitabine, and simultaneously reduces the 5′-nucleotidase, responsible for deactivation of gemcitabine, suggesting a possible additional role of these enzymes in the enhanced cytotoxic activity of gemcitabine. Finally, a significant *in vivo* antitumour effect on MIAPaCa-2 xenografts was observed with the simultaneous combination of fluvastatin and gemcitabine, resulting in an almost complete suppression and a marked delay in relapse of tumour growth. In conclusion, the combination of fluvastatin and gemcitabine is an effective cytotoxic, proapoptotic treatment *in vitro* and *in vivo* against MIAPaCa-2 cells by a mechanism of action mediated, at least in part, by the inhibition of p21*ras* and *rhoA* prenylation. The obtained experimental findings might constitute the basis for a novel translational research in humans.

Pancreas cancer represents a model of carcinogenesis in which the mutational activation of the k-*ras* oncogene is present in up to 90% of cases, and is supposed to play a relevant role in tumour progression and aggressive behaviour (Cowgill and Muscarella, 2003). Small GTP-binding proteins of the Ras superfamily, including Rho, Rab, Raf, Rac and Rap, are involved in diverse cellular functions such as cytokinesis, cell motility, cell adhesion and cell proliferation (Etienne-Manneville and Hall, 2002). Signal transduction is mediated through the activation of the p21*ras* as a consequence of GTP binding to the protein and through the activation of mitogen-activated protein kinases (MAPKs) belonging to the serine/threonine protein kinase family (Kolch, 2002). Before either *ras* and *ras*-related proteins can be biologically active, they undergo isoprenylation at the COOH-terminal CAAX motif (Liang *et al*, 2002). Recently, the 3-hydroxy-3-methylglutaryl-coenzyme A (HMG-CoA) reductase inhibitors have received great attention for their cholesterol-independent (pleiotropic) effects, such as the possible inhibition of small GTP-binding proteins Rho, Ras and Rac isoprenylation (Liao, 2002). Moreover, preclinical data seem to suggest an important role of statins as pharmacological tools for controlling abnormal cell growth, such as myocyte or cancer cell proliferation (Kaushal *et al*, 2003); lovastatin and other statins suppressed the proliferation of numerous cancer cell lines, including human pancreas cancer cells, and exhibited a trigger activity in tumour-specific apoptosis (Wong *et al*, 2002). Holstein and Hohl (2001b) demonstrated a synergistic interaction between paclitaxel or cytosine arabinoside (Holstein and Hohl, 2001a) and lovastatin on human cancer cell lines, whereas Feleszko *et al* showed a potentiated antitumour activity of cisplatin (Feleszko *et al*, 1998) and doxorubicin (Feleszko *et al*, 2000) in murine tumour models when associated with lovastatin. Fluvastatin, the first entirely synthetic hydrophylic HMG-CoA reductase inhibitor, is presently used for the treatment of patients with hypercholesterolaemia (Lawrence and Reckless, 2002). It has similar efficacy and tolerability profiles than other HMG-CoA reductase inhibitors, but, unlike them, it has not been associated with rhabdomyolysis and myopathy (Scripture and Pieper, 2001). Few reports have been focused on fluvastatin inhibition of cancer cell proliferation (Seeger *et al*, 2003) and its possible mechanism of action (Kusama *et al*, 2001, 2002). To our knowledge, no combination studies have been performed with currently available chemotherapeutic drugs in order to test a hypothetic synergism with their anticancer activity and the molecular basis of the eventual positive interaction. The cytosine arabinoside analogue 2′,2′-difluorodeoxycytidine (gemcitabine) has been proven to be active in the treatment of pancreatic cancer (Abbruzzese, 2002a) with significant clinical benefit, but still with marginal survival advantage (El Rayes *et al*, 2003). Gemcitabine inhibits cell growth by interfering with the synthesis of DNA (Barton-Burke, 1999), and efforts are currently being made to increase the therapeutic efficacy of the drug in clinical settings on various types of cancer by combination with other agents, including cisplatin, oxaliplatin, irinotecan, docetaxel, 5-fluorouracil, capecitabine or pemetrexed (Heinemann, 2002; Jacobs, 2002). Moreover, numerous preclinical experimental studies have been made to enhance the antitumour effects of gemcitabine using novel therapeutic approaches such as the proteasome inhibitor bortezomib (Kamat *et al*, 2004) and the antiangiogenic drug SU5416 (Bocci *et al*, 2004).

Gemcitabine and fluvastatin do not have overlapping toxicities; therefore, the association of these compounds might be an attractive clinical alternative for the treatment of advanced pancreatic tumours. The purposes of this study are to determine: (1) the antiproliferative, proapoptotic effects of fluvastatin on *k*-ras-mutated MIAPaCa-2 human pancreatic cancer cells and its probable mechanism of action; (2) the synergistic enhancement of cytotoxicity by the combination with gemcitabine; (3) the possible underlying molecular basis of the synergism with pharmacological tools such as PD98059, a MEK1/2 inhibitor that can block activation of downstream ERK-1/2, and the expression of genes such as the deoxycytidine kinase (dCK), a rate-limiting enzyme required for the activation of gemcitabine, and the 5′-nucleotidase (5′-NT), responsible for deactivation of gemcitabine; (4) the *in vivo* effects of the fluvastatin/gemcitabine combination on MIAPaCa-2 xenografts in nude mice.

## MATERIALS AND METHODS

### Materials and animals

Antipain, leupeptin, aprotinin, sodium dodecyl sulphate (SDS) and proteinase K were obtained from Roche Molecular Biochemicals (Mannheim, Germany). DMEM medium, foetal bovine serum (FBS), foetal calf serum (FCS), horse serum (HS), L-glutamine, penicillin, streptomycin, agarose and 180 bp DNA ladder were from Gibco (Gaithersburg, MD, USA), swine serum was from Dako (Milan, Italy) and acrylamide was purchased from Bio-Rad (Melville, NY, USA). Diethylamine (DEA) and Nonidet P-40 were obtained from ICN Biomedicals Inc. (Costa Mesa, CA, USA); mouse IgG1 anti-p21*ras* monoclonal antibody was purchased from Transduction Laboratories (Lexington, KY, USA), whereas anti-p21*rhoA* and anti-p42MAPK/ERK2 rabbit polyclonal antibodies were from Santa Cruz Biotechnology Inc. (Santa Cruz, CA, USA). Horseradish peroxidase-conjugated secondary antibodies and reagents for chemiluminescence detection of proteins in immunoblots were purchased from Amersham Life Science (ECL Western detection kit, Little Chalfont, UK). Universal Mount was from Research Genetics Inc. (Huntsville, AL, USA). Quantitative real-time polymerase chain reaction (PCR) reagents were purchased from Applied Biosystems (Foster City, CA, USA). All other chemicals not listed in this section were obtained from Sigma Chemical Co. (St Louis, MO, USA). Fluvastatin (Novartis, Basel, Switzerland) and gemcitabine (Ely Lilly and Company, Indianapolis, IN, USA) were dissolved in sterile distilled water, then diluted in sterile culture medium immediately before their *in vitro* use, or in sterile saline solution for *in vivo* use. Plastic for cell culture was supplied by Costar (Cambridge, MA, USA). PD98059 was purchased from Calbiochem Biochemicals (Milano, Italy), dissolved in DMSO and diluted in culture medium.

The CD nu/nu male mice, weighing 20–25 g, were supplied by Charles River (Milan, Italy) and were allowed unrestricted access to food and tap water. Housing and all procedures involving animals were performed according to the protocol approved (approval number 11/04) by the ‘*Comitato di Ateneo per la sperimentazione animale*’ (Academic Committee for the animal experimentation) of the University of Pisa, in accordance with the European Community Council Directive 86-609, recognised by the Italian government, on animal welfare and the guidelines of the UK Co-ordination Committee on Cancer Research (UKCCCR). Each experiment employed the minimum number of mice needed to obtain statistically meaningful results.

### Cell culture conditions

The human pancreatic cancer cell line MIAPaCa-2 was obtained from the American Type Culture Collection (ATCC, Manassas, VA, USA). MIAPaCa-2 cells were maintained in DMEM medium, supplemented with 10% FBS, 2.5% HS, penicillin (50 IU ml^−1^), streptomycin (50 *μ*g ml^−1^) and L-glutamine (2 mM). The human colon cancer cell line COLO320-DM, a cell line with wild-type K-*ras* (Di Paolo *et al*, 2000), was from ATCC and maintained in RPMI 1640 medium supplemented with 10% FBS, penicillin and L-glutamine. Cells were routinely grown in 75 cm^2^ tissue culture flasks and kept in a humidified atmosphere of 5% CO_2_ at 37°C. Cells were harvested with a solution of 0.25% trypsin–0.03% EDTA when they were in log phase of growth, and maintained at the above-described culture conditions for all experiments.

### Polymerase chain reaction analysis of K-*ras* sequence in the MIAPaCa-2 cell line

In order to demonstrate a possible mutation of K-*ras* sequence, the mutational analysis of the codon 12 of the K-*ras* gene was performed in MIAPaCa-2 cells by oligodeoxynucleotide hybridisation, as described previously (Marchetti *et al*, 1996). Briefly, as a negative control the CLONE cell line (ATCC), derived from a normal human corneal epithelium, was analysed, while as a positive control a sample of human lung adenocarcinoma with mutation at codon 12 (GGT → TGT, Gly → Cys) was used. The primers used to amplify the K-*ras* gene around codon 12 were: 5′-GGCCTGCTGAAAATGACTGA-3′ and 5′-TGATTCTGAATTAGCTGTAT-3′. The amplified products of the PCR were denatured, blotted onto nylon membranes and then hybridised with ^32^P-labelled oligonucleotide probes designed to detect *ras* mutations.

### Cytotoxicity assay

*In vitro* chemosensitivity testing was performed on single-cell suspensions of MIAPaCa-2 cells (2 × 10^4^ cells well^−1^) plated in six-well sterile plastic plates and allowed to attach overnight. The treatment protocol was designed so that each drug concentration was represented by at least nine wells. Cells were treated with gemcitabine (1–500 nM) or PD98059 (1–100 *μ*M), or fluvastatin (0.1–20 *μ*M) for 72 h with or without mevalonic acid 100 *μ*M; in separate experiments, cells received drugs either simultaneously or sequentially as described below. Furthermore, in order to test fluvastatin antiproliferative activity on a wild-type k-*ras* cancer cell line, 2 × 10^4^ COLO320-DM cells per well were treated with fluvastatin (0.1–100 *μ*M) for 72 h. At the end of the experiment, cells were photographed with a phase-contrast microscope Leitz MD IL (Leica, Heerbrugg, Switzerland) and then washed with PBS, harvested with trypsin/EDTA, and counted with a haemocytometer. The survival of treated cells was expressed as a percentage of control (vehicle treated) cultures. The concentration of drugs that reduced cell survival by 50% (IC_50_) as compared to controls was calculated. Fluvastatin combined with gemcitabine was explored with three different treatment schedules at a fixed molar concentration ratio of 100 : 1 in MIAPaCa-2 cells, as follows: (A) *simultaneous exposure*: fluvastatin (0.1–20 *μ*M) plus gemcitabine (1–200 nM) for 72 h; (B) *sequential exposure*: fluvastatin (0.1–20 *μ*M) alone for 24 h, fluvastatin (0.1–20 *μ*M) plus gemcitabine (1–200 nM) for 24–72 h and gemcitabine alone for 72–96 h; (C) *reverse exposure*: gemcitabine (1–200 nM) alone for 24 h, gemcitabine (1–200 nM) plus fluvastatin (0.1–20 *μ*M) for 24–72 h and fluvastatin (0.1–20 *μ*M) alone for 72–96 h. Therefore, the total exposure of each drug was 72 h. After drug exposure, the media of cell cultures were discarded and fresh medium was supplied to cells. Furthermore, PD98059 (0.1–5 *μ*M) and gemcitabine (1–50 nM) were administered simultaneously for 72 h to the pancreatic cells at a fixed molar concentration ratio of 100 : 1.

### Assessement of synergism or antagonism

To evaluate the level of interaction (synergistic, additive or antagonist) between gemcitabine and fluvastatin or PD98059, the method proposed by Chou *et al* (1993) was followed. Briefly, synergism or antagonism for gemcitabine plus fluvastatin or PD98059 is calculated on the basis of the multiple drug-effect equation, and quantitated by the combination index (CI), where CI<1, CI=1 and CI>1 indicate synergism, additive effect and antagonism, respectively. Based on the classic isobologram, the CI value is calculated as: 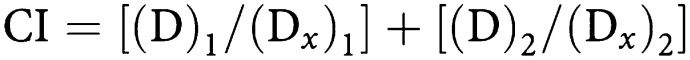


At the 75% inhibition level, (D_*x*_)_1_ and (D_*x*_)_2_ are the concentrations of gemcitabine and fluvastatin or PD98059, respectively, that induce a 75% inhibition of cell growth; (D)_1_ and (D)_2_ are the concentrations of gemcitabine and fluvastatin or PD98059 in combination that also inhibits cell growth by 75% (isoeffective as compared with the single drugs alone). The dose-reduction index (DRI) defines the degree of dose reduction that is possible in combination for a given degree of effect as compared with the concentration of each drug alone:
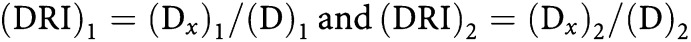


### Quantitative, real-time PCR analysis of dCK and 5′-NT gene expression

To evaluate the expression of the dCK, a rate-limiting enzyme required for the activation of the pyrimidine analogue gemcitabine, and of the cytosolic 5′-NT, responsible for deactivation of nucleotides and of the activated gemcitabine (Danesi *et al*, 2003), MIAPaCa-2 cells were treated with fluvastatin (1 and 5 *μ*M) or vehicle alone for 72 h. Quantitative real-time PCR analysis was performed as described previously (Giovannetti *et al*, 2005). Briefly, RNA (1 *μ*g) was reverse transcribed at 37°C for 1 h in a 100-*μ*l reaction volume containing 0.8 mM deoxynucleotide mix (dNTPs), 200 U of Moloney murine leukaemia virus reverse transcriptase (MMLV-RT), 40 U of RNase inhibitor and 0.05 *μ*g ml^−1^ random primers. The resulting cDNA was diluted (2 : 3) and then amplified by QRT-PCR with the Applied Biosystems 7900HT sequence detection system (Applied Biosystems). Polymerase chain reaction thermal cycling conditions, design and optimisation of primer concentrations were reported in detail by Giovannetti *et al* (2005). Amplifications were normalised to glyceraldehyde 3-phosphate dehydrogenase (GAPDH), and the quantitation of gene expression was performed using the ΔΔ*C*_t_ calculation, where *C*_t_ is the threshold cycle; the amount of target, normalised to the endogenous control and relative to the calibrator (untreated control cells), is given as 
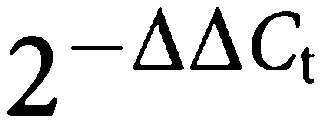
.

### Assay of apoptosis

The internucleosomal DNA fragmentation was assayed as reported (Danesi *et al*, 1995), with minor modifications. Briefly, MIAPaCa-2 cells were plated in 100 mM sterile dishes for cell culture and treated for 72 h with gemcitabine 20–200 nM, fluvastatin 0.5–20 *μ*M alone or in combination with mevalonic acid 100 *μ*M, or their simultaneous combination at a fixed concentration ratio of 1 : 100 of gemcitabine/fluvastatin. Furthermore, in order to test fluvastatin proapoptotic activity on a wild-type k-*ras* cancer cell line, COLO320-DM cells were treated with fluvastatin (3–50 *μ*M) for 72 h. At the end of incubation, 2 × 10^6^ cells per treatment were washed with PBS, harvested with trypsin-EDTA and pelleted by centrifugation. Cells were lysed in Tris-EDTA buffer for 90 min at 4°C. Cellular lysates were centrifuged at 15 000 r.p.m. for 1 h at 4°C, and clear supernatants containing fragmented chromatin were incubated at 42°C for 30 min in the presence of proteinase K (200 *μ*g ml^−1^), and extracted by treatment with phenol : chloroform : isoamyl alcohol (25 : 24 : 1), vigorously shaken for 30 s and centrifuged at 15 000 r.p.m. for 10 min. Supernatants were collected and mixed with 100 *μ*l of NaCl 5 M, 1 ml of cold 100% ethanol and 1 *μ*l of glycogen. The suspension were kept at −20°C overnight to precipitate DNA fragments, then centrifuged at 15 000 r.p.m. for 30 min. Pellets were washed with 70% ethanol and dried under air flow. Samples were resuspended in Tris-EDTA buffer containing 1 mg ml^−1^ boiled bovine pancreatic RNAse A, incubated for 1 h at 40°C, and finally mixed with DNA sample buffer. Electrophoresis was performed in 1% agarose gel in Tris-EDTA-acetate buffer, and bands were visualised by ethidium bromide staining and UV transillumination. A 123-bp DNA ladder was run as a standard. Gel was photographed with a Polaroid MP4 Land camera (Polaroid, Cambridge, MA, USA) and pictures were digitised for the analysis of DNA fragmentation as described in the section ‘Analysis of data’.

### Cell cycle analysis

Cells were plated in 100 mm sterile dishes for cell culture and treated for 72 h with gemcitabine 1–50 nM or fluvastatin 0.1–10 *μ*M. At the end of the experiment, cells were washed with PBS and harvested with EDTA (0.5 mM), centrifuged, resuspended in PBS and counted. Flow cytometry analysis of DNA content was performed in 75% ethanol-fixed cells, washed twice in PBS/0.1% NaN_3_/1% FCS and filtrated through a 35 *μ*m nylon gauze filter; 1 × 10^6^ cells ml^−1^ were stained for 30 min at 4°C in the dark with 50 *μ*g ml^−1^ propidium iodide solution containing 100 U ml^−1^ RNAse and 0.1% Nonidet P-40. Flow cytometry analysis was performed on FACSort flow cytometry apparatus (Becton Dickinson, San Jose, CA, USA), equipped with a laser for excitation at 488 nM. CellQuest version 3.1 and ModFit LT2 version 2.0 (Verity Software House Inc., ME, USA) softwares were used for analysis of cell cycle distribution and quantitation of S-phase fraction on at least 20 000 nuclei. Clumped cells, doublets and higher aggregates were gated out using electronic pulse-processing (peak *vs* integrated DNA fluorescence signals) and measurement accuracy was evaluated considering the coefficient of variation (CV) of the G_0_/G_1_ peaks.

### Immunoblot analysis of p21*ras*, p21*rhoA* and p42MAPK/ERK2

Total cellular extracts were analysed for p21*ras* and p21*rhoA* by immunoblotting. Briefly, cells exposed to fluvastatin (2–10 *μ*M) or gemcitabine (1–50 nM) for 72 h alone or in combination with mevalonic acid 100 *μ*M and untreated controls were harvested with EDTA 0.5 mM, washed with PBS and centrifuged. The cells were solubilised at 4°C in lysis buffer (Tris-base 50 mM, pH 7.6, 2 mM EDTA, 100 mM NaCl, 1% Nonidet P-40, 1 mM PMSF, 2 *μ*g ml^−1^ aprotinin, 2 *μ*g ml^−1^ pepstatin, 2 *μ*g ml^−1^ antipain) for 60 min. Cell lysate was centrifuged at 15 000 r.p.m. for 20 min at 4°C and aliquots of supernatants were used to measure protein concentration. Samples were boiled for 3 min in SDS-sample buffer (50 mM Tris-base, pH 6.8, 2% SDS, 100 mM dithiothreitol, 10% glycerol and 0.025% *β*-mercaptoethanol) and separated on 15% SDS–polyacrylamide gel electrophoresis (SDS–PAGE). Proteins were then transferred onto Immobilon-P membrane (Millipore, Bedford, MA, USA) and blots were probed with an anti-p21*ras* (1 : 500) or anti-p21*rhoA* antibody (1 : 1000), and detected with the use of horseradish peroxidase-conjugated secondary antibody (dilution, 1 : 10 000). The membranes were then exposed to Kodak X-Omat AR film, and film densities were quantified as described in ‘Analysis of data’. To evaluate the effect of fluvastatin and gemcitabine on p42MAPK/ERK2 phosphorylation, MIAPaCa-2 cells were treated with fluvastatin (2–10 *μ*M) or gemcitabine (1–50 nM) for 72 h alone or in combination with mevalonic acid 100 *μ*M. Cells were solubilised in lysis buffer with the protein phosphatase inhibitors sodium metavanadate and sodium fluoride (200 *μ*M each) for 45 min at 4°C, and then centrifuged at 4°C for 20 min at 15 000 r.p.m. The supernatant was boiled for 3 min in SDS-sample buffer and separated on 11% SDS–PAGE. Proteins were transferred onto Immobilon-P membrane and probed with anti-p42MAPK/ERK2 antibody (1 : 1000), and detected as described above.

### Immunocytochemistry

Cells were seeded onto chamberslides (Nalge Nunc, Naperville, IL, USA) at a density of 10^4^ per 150 *μ*l; after treatment with fluvastatin at 2 *μ*M for 48 h or vehicle, cells were washed in PBS and fixed with 1% neutral buffered formalin (10 min) at 4°C. The specimens were then permeabilised with a 10-min exposure to a solution of 0.2% Triton X-100–PBS. The samples were subsequently treated with a solution of 3% H_2_O_2_ for 5 min to quench endogenous peroxidase activity, and nonspecific reactivity was blocked with 5% swine serum for 20 min at 37°C. The samples were then incubated overnight at 4°C in a humidified chamber with the mouse anti-human-p21*rhoA* or anti-human-Ha-p21*ras* antibody (1 : 20–1 : 50 in 0.1% BSA-PBS). The detection protocol was carried out using biotinylated secondary antibodies and a streptavidin–peroxidase complex (LSAB kit, Dako). The reaction was developed by incubating samples in the substrate–chromogen solution (1 mg ml^−1^ 3,3′-diaminobenzidine tetrahydrochloride containing 0.02% H_2_O_2_) for 5 min in the dark. Finally, the slides were mounted with Universal Mount and observed with a DMRB Leica microscope equipped with a × 100 oil immersion lens. Each treatment was performed in three wells and observations were made from 10 fields from each well. Negative controls were obtained by substituting the primary antibodies with a nonimmune mouse serum or with 1 × PBS+0.1% BSA, or by using primary antibodies not raised against p21*rhoA* or Ha-p21*ras* (e.g., anti-human TGF*α* immunoglobulins; Oncogene Science, Uniondale, USA). Endogenous peroxidase and avidin-binding activity were tested by incubating the slides with 3,3′-diaminobenzidine tetrahydrochloride alone or with streptavidin–HRP complex alone, followed by 3,3′-diaminobenzidine tetrahydrochloride, respectively.

### *In vivo* studies

MIA PaCa-2 cell viability was assessed by trypan blue dye exclusion, and, on day 0, 1.3 × 10^6^±5% cells per mouse were inoculated subcutaneously between the scapulae in 0.2 ml per mouse of culture medium without FBS using an insulin syringe with a 0.5 × 16 mm needle. Animal weights were monitored and the appearance of a subcutaneous tumour that was measured every 2 days in two perpendicular directions using calipers. Tumour volume (mm^3^) was defined as follows: ((*w*_1_ × *w*_2_ × *w*_2_) × (*π*/6)), where *w*_1_ and *w*_2_ were the largest and the smallest tumour diameters (mm), respectively. The mice were randomised into groups of five. In order to treat an established tumour (∼35 mm^3^), at day 15 from the cell inoculum, fluvastatin, gemcitabine or their simultaneous combination was administered intraperitoneally as follows: (1) fluvastatin every 2 days at a dose of 30 mg kg^−1^ for 14 days (cumulative dose of 210 mg kg^−1^ per mouse equivalent to the study by Ferrara *et al*, 2003); (2) gemcitabine 120 mg kg^−1^ four times at 3-day intervals as described previously (Braakhuis *et al*, 1995); (3) combination treatment of fluvastatin and gemcitabine. The control group was injected i.p. with vehicle alone (saline solution). The experimental period ended 11 days after the last injection of fluvastatin and mice were killed by an anaesthetic overdose.

### Analysis of data

Film densities of protein immunoblots and apoptosis assays were quantified through video imaging densitometry with the KS300 version 1.2. software (Kontron Elektronic, Eching, Germany), and expressed as arbitrary units of mean gray values (optical density), in the range of 0–255, where 0 was black and 255 was white (Di Paolo *et al*, 2000). Results were expressed as the mean±s.e. of the optical density ratio between the gray values of isoprenylated and nonisoprenylated proteins for p21*rhoA* and p21*ras*, and between the nonphosphorylated and phosphorylated immunoblot bands for p42ERK2/MAPK. The degree of apoptosis was assessed by single-band densitometric analysis of DNA fragments in the range of 180–900 bp. The analysis by ANOVA, followed by the Student–Newman–Keuls test, was used to assess the statistical differences of data obtained in control and treated cells with respect to immunoblotting, cytotoxicity, real-time RT–PCR results and *in vivo* studies. *P*-values lower than 0.05 were considered significant.

## RESULTS

### Presence of K-*ras* gene mutation in the MIAPaCa-2 cell line

**Figure 1:**
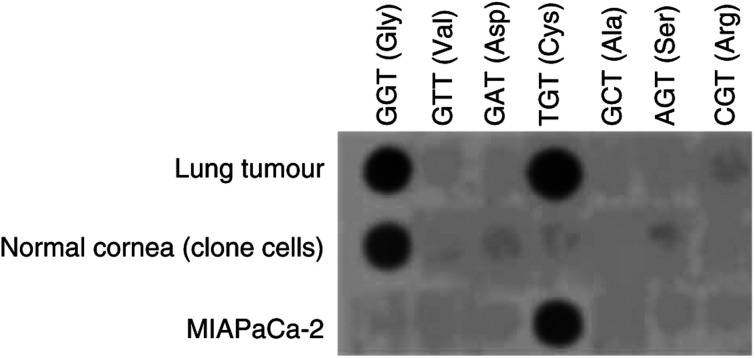
Dot-blot hybridisation analysis of point mutations (GGT → TGT) at codon 12 of the K-ras oncogene in MIAPaCa-2 and lung tumour; normal cornea (clone cell line) shows wild-type K-ras.

The analysis of DNA extracted from MIAPaCa-2 cells demonstrated a homozygous GGT → TGT mutation at codon 12 of K-*ras* gene, while one allele of the lung tumour sample was mutated; cells from normal cornea showed two normal alleles (Figure 1).

### Cytotoxicity of gemcitabine and fluvastatin

**Figure 2:**
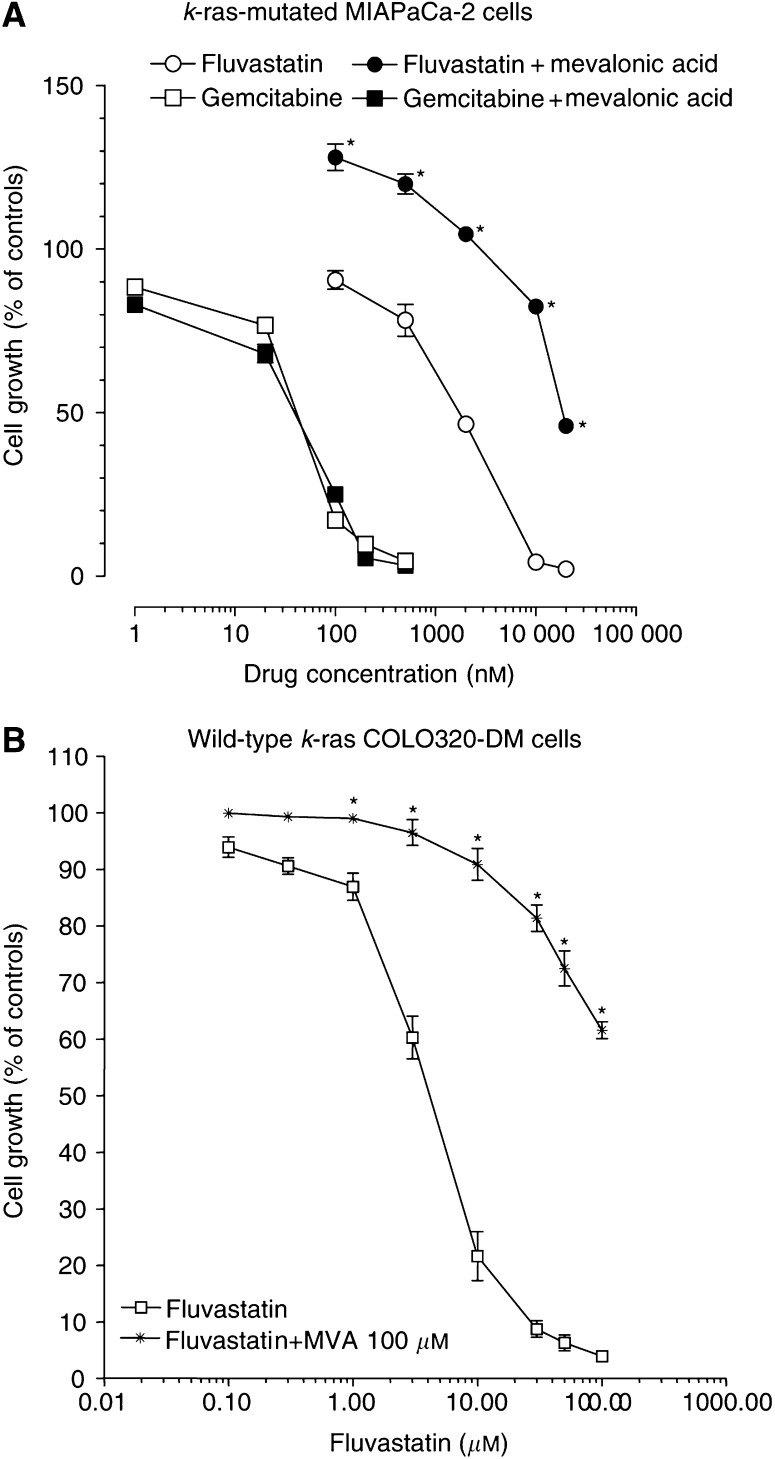
(**A**) Effect of fluvastatin, gemcitabine and their combination with mevalonic acid 100 *μ*M on *k*-ras-mutated MIAPaCa-2 cell proliferation; (**B**) effect of fluvastatin and its combination with mevalonic acid 100 *μ*M on wild-type *k*-ras COLO320-DM cell proliferation. Symbols and bars, mean values±s.e., respectively; ^*^P<0.05 *vs* fluvastatin only.

Gemcitabine and fluvastatin inhibited cell growth of MIAPaCa-2 cell line in a concentration-dependent manner (Figure 2A), and the IC_50_ values were 19.7±0.4 nM and 1.07±0.27 *μ*M, respectively. The cytotoxic activity of gemcitabine 1–500 nm was not modified by mevalonic acid 100 *μ*M (IC_50_=13.8±1.2 nM; Figure 2A), while the antiproliferative effect of fluvastatin 0.1–20 *μ*M was greatly reduced and reversed by adding mevalonic acid 100 *μ*M in the medium (Figure 2A), as demonstrated by the IC_50_ value (IC_50_=18.83±1.2 *μ*M) being significantly different from that observed in the absence of mevalonic acid (*P*<0.05). Moreover, the PD98059 72-h treatment showed an antiproliferative effect on serum-stimulated MIAPaCa-2 cells, as demonstrated by the experimental IC_50_ of 6.13±0.8 *μ*M. In order to further test the antiproliferative activity of fluvastatin on a wild-type *k*-ras cancer cell line, COLO320-DM cells were chosen. Fluvastatin inhibited COLO320-DM cell proliferation in a dose-dependent manner (Figure 2B), and the calculated IC_50_ value was 4.90±0.68 *μ*M. The cytotoxic activity of fluvastatin on COLO320-DM cell growth was significantly reduced by a concentration of 100 *μ*M mevalonic acid (Figure 2B).

**Figure 3:**
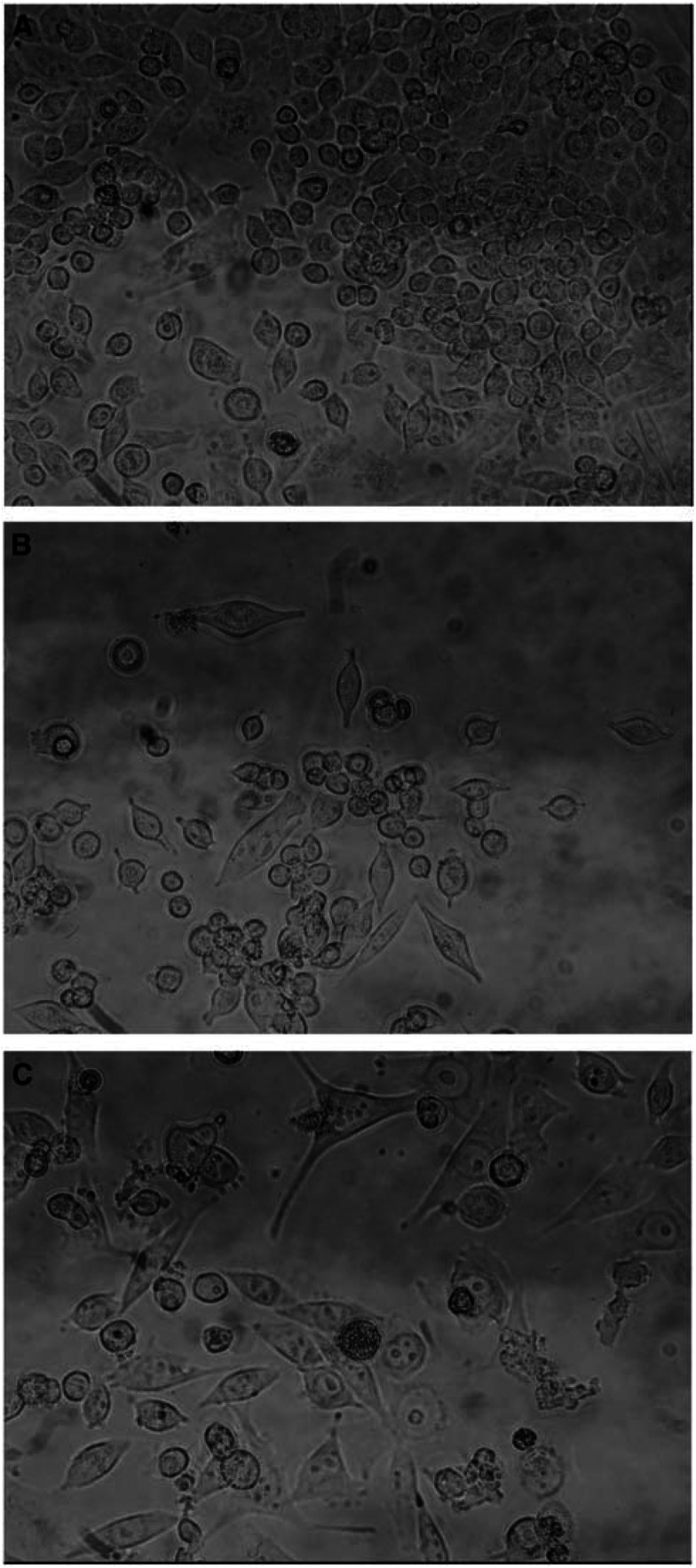
Microscopic pictures of control MIAPaCa-2 cells (**A**) and cells treated with fluvastatin 2 *μ*M (**B**) or gemcitabine 20 nM (**C**). Original magnification × 100.

**Figure 4:**
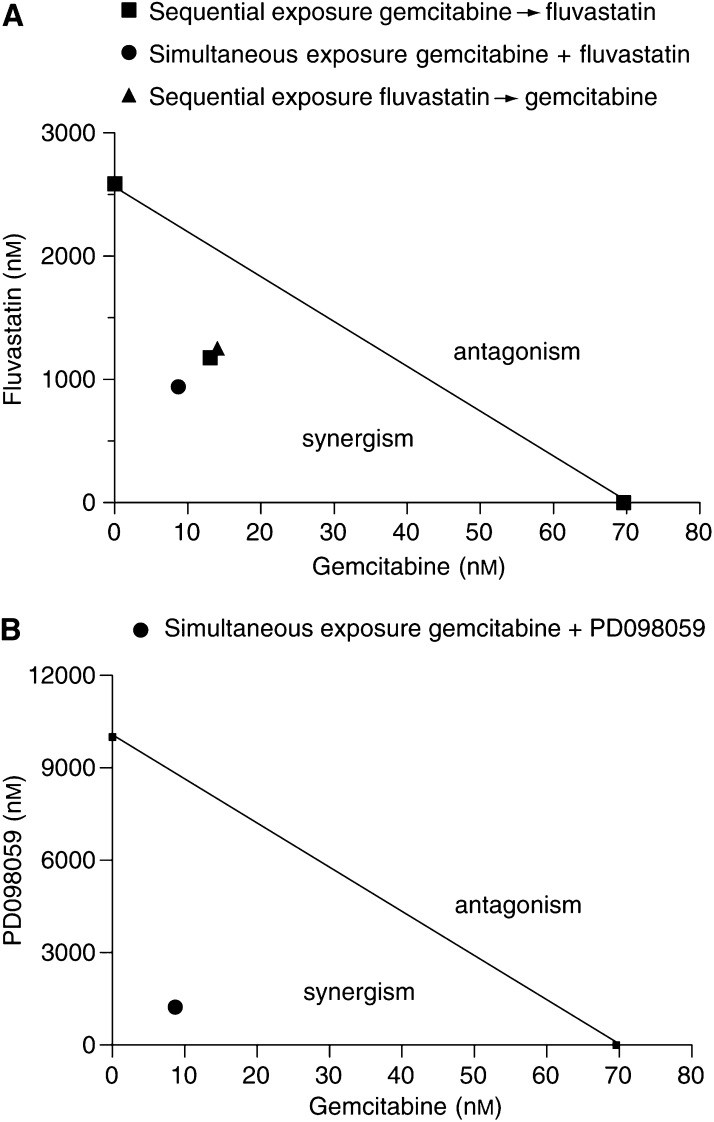
Isobologram analysis of MIAPaCa-2 cell growth inhibition by (**A**) gemcitabine and fluvastatin simultaneously and sequentially, and (**B**) gemcitabine and PD098059 simultaneously. The IC_75_ values of each drug are plotted on the axes; the solid line represents the addictive effect, while the points representing the concentrations of gemcitabine and fluvastatin or PD098059 resulting in 75% growth inhibition of the combination are reported on the left of the connecting line, indicating synergism.

From a morphological point of view, MIAPaCa-2 cells treated with vehicle alone grew in large colonies as confluent monolayers with an epithelial-like shape (Figure 3). Most cells treated with fluvastatin 2 *μ*M for 72 h became rounded and the proportion of floating cells increased in a concentration-dependent manner (Figure 3); also, gemcitabine 20 nM determined marked aspecific degenerative alterations of cell shape after 72 h (Figure 3). Simultaneous and sequential exposure to fluvastatin and gemcitabine showed synergism at effect levels exceeding 60% inhibition (Table 1). Synergism corresponding to CI<1 always yielded a favourable DRI (>1) for both drugs (Tables 1 and 2). The DRI values at IC_50_, IC_75_ and IC_95_ are reported in Table 2. Figure 4A shows a representative isobologram of MIAPaCa-2 cells exposed to gemcitabine and fluvastatin for 72 h with different schedules of treatment. Furthermore, the simultaneous treatments of gemcitabine and PD98059 for 72 h were synergistically active on pancreatic cell proliferation, as shown by a representative isobologram in Figure 4B. The position of the data points on the left of the line connecting the IC_75_ values of gemcitabine and fluvastatin or PD98059 indicates synergism for all schedules (Figure 4A and B).

### dCK and 5′-NT gene expression in fluvastatin-treated cancer cells

**Figure 5:**
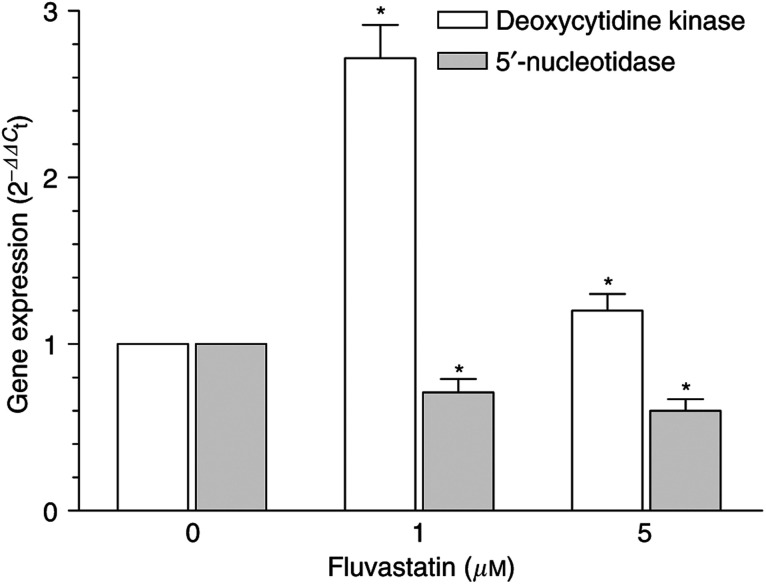
Deoxycytidine and 5′-NT expression in fluvastatin-treated MIAPaCa-2 cancer cells. Columns, mean values obtained from three independent experiments; bars, ±s.e.; ^*^, statistically different from control cells (*P*<0.05).

Fluvastatin, at its 1 and 5 *μ*M concentrations, significantly increased dCK expression (e.g. at 1 *μ*M, 271.6 *vs* 100% of controls) in the MIAPaca-2 cell line, whereas, at the same concentrations, there was a significant decrease of 5′-NT expression (e.g. at 1 *μ*M, 71.1 *vs* 100%; Figure 5). Thus, both the simultaneous significant increase in the expression of the activating enzyme (dCK) and the decrease of the deactivating one (5′-NT) suggested a possible role of fluvastatin in the activating metabolism of gemcitabine in pancreatic cancer cells.

### Induction of apoptosis by gemcitabine, fluvastatin and their combination

**Figure 6:**
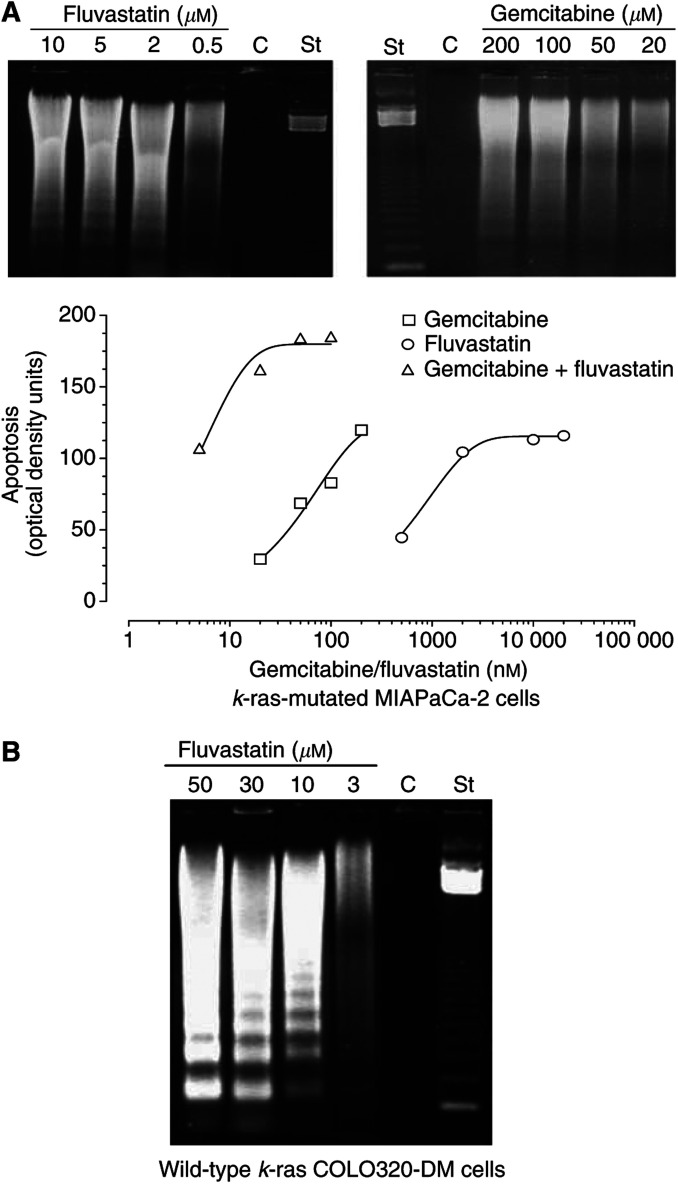
(**A**) Gel electrophoresis of DNA extracted from fluvastatin- and gemcitabine-treated *k*-ras-mutated MIAPaCa-2 cells (upper). Image analysis of apoptotic DNA from cells exposed to fluvastatin, gemcitabine and their combination (lower); (**B**) gel electrophoresis of DNA extracted from 72 h fluvastatin-treated wild-type *k*-ras COLO320-DM cells. C, control; St, standard ladder.

The extent of DNA fragmentation was dependent on the concentration of both drugs (Figure 6A). In particular, the production of chromatin fragments was clearly detectable after 72 h in a dose-dependent manner for fluvastatin and gemcitabine (Figure 6A). The use of mevalonic acid 100 *μ*M determined a complete reversion of the apoptosis induced by fluvastatin, but not by gemcitabine, on MIAPaCa-2 cells (data not shown). Image analysis of DNA fragmentation confirmed that the increase in drug concentrations was associated with enhanced optical density of DNA bands corresponding to shorter fragments (180–900 bp; Figure 6A). A plateau was reached at the highest concentration of fluvastatin; finally, the simultaneous treatment of MIAPaCa-2 cells with fluvastatin/gemcitabine at the 100 : 1 concentration ratio was associated with a marked increase in apoptosis (Figure 6A). The concentration-dependent proapoptotic effects of 72 h fluvastatin treatment were confirmed also in the wild-type *k*-ras COLO320-DM cancer cell line (Figure 6B).

### Cell-cycle-specific effects of gemcitabine and fluvastatin

**Figure 7:**
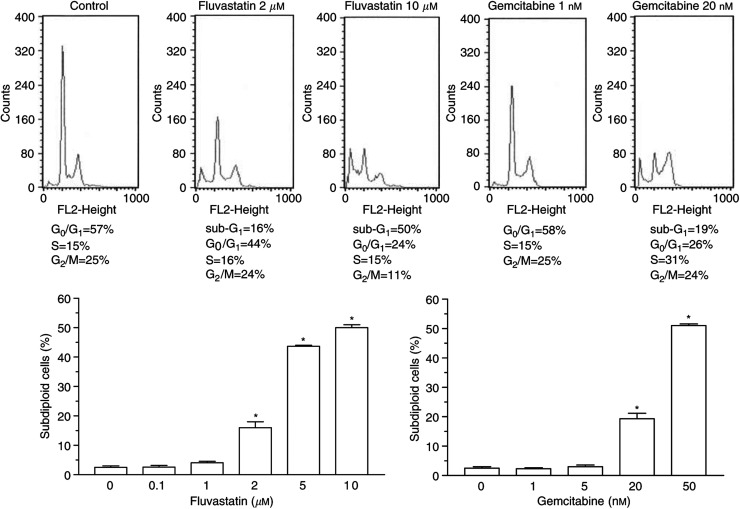
*In vitro* effect of fluvastatin and gemcitabine on cell cycle distribution of MIAPaCa-2 cells. Percent values of cells in different phases of the cell cycle are given in each panel. The subdiploid peak in DNA histograms from treated cells indicates the occurrence of apoptosis (upper). The amount of subdiploid, apoptotic cells raises as the concentrations of the drugs in the culture media increase (lower). Columns and bars, mean values±s.e., respectively. ^*^P<0.05 *vs* controls.

The DNA histograms obtained from MIAPaCa-2 cells treated with fluvastatin or gemcitabine showed a dose-dependent drug effect on the cell cycle distribution (Figure 7). When the treatment with fluvastatin was prolonged up to 72 h, the G_2_/M peak declined concomitantly to the appearance of a dose-dependent subdiploid peak (sub G_1_), typical of apoptotic cells, starting from a concentration of 2 *μ*M (Figure 7). Gemcitabine induced an S-phase accumulation of cells after 72 h of treatment, with the simultaneous appearance of the subdiploid peak, starting at 20 nM (Figure 7). Apoptosis was quantified and measured as the percentage of subdiploid cells on the DNA histogram. Both fluvastatin and gemcitabine caused a significant dose-dependent increase in apoptosis (*P*<0.05; Figure 7), thus confirming the data obtained from agarose gel electrophoresis.

### Inhibition of p21*rhoA* and p21*ras* prenylation and phosphorylation of p42MAPK/ERK2 by fluvastatin

**Figure 8:**
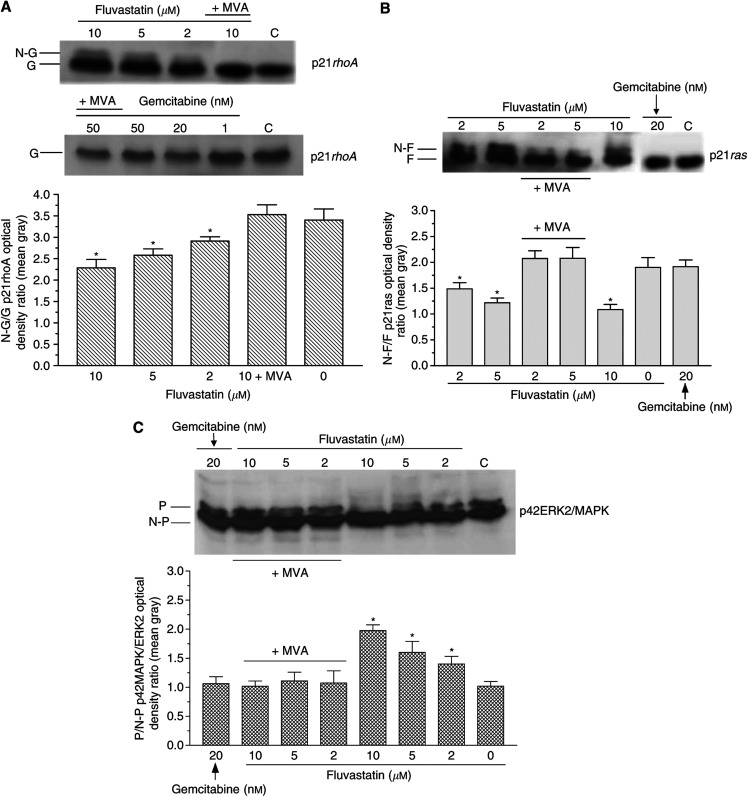
(**A**) Immunoblotting (upper) and image analysis (lower) of p21*rhoA* from total cellular lysates of MIAPaCa-2 cells treated with fluvastatin and gemcitabine. Columns and bars, mean values±s.e., respectively. MVA, mevalonic acid, G, geranyl-geranylated, N-G, nongeranyl-geranylated p21*rhoA*; ^*^P<0.05 *vs* controls. (**B**) Immunoblotting (upper) and image analysis (lower) of p21*ras* from total cellular lysates of MIAPaCa-2 cells treated with fluvastatin and gemcitabine. Columns and bars, mean values±s.e., respectively. MVA, mevalonic acid, F, farnesylated, N-F, nonfarnesylated p21*ras*; ^*^P<0.05 *vs* controls. (**C**) Immunoblotting (upper) and image analysis (lower) of p42MAPK/ERK2 in MIAPaCa-2 cells treated with fluvastatin and gemcitabine. Columns and bars, mean values±s.e., respectively. MVA, mevalonic acid, P, phosphorylated, N-P, nonphosphorylated p42MAPK/ERK2; ^*^P<0.05 *vs* controls.

Immunoblots of MIAPaCa-2 cells demonstrated that fluvastatin inhibited the post-translational processing of immature p21*rhoA*, causing the appearance of nongeranyl-geranylated p21*rhoA* proportional to the drug concentrations (Figure 8A). The image analysis of protein bands, computed as the ratio between the mean gray values of the prenylated *vs* nonprenylated band on Western blots of p21*rhoA*, confirmed that fluvastatin significantly increased the amount of immature, nonisoprenylated protein in a concentration-dependent manner (Figure 8A). The cells treated simultaneously with fluvastatin and 100 *μ*M mevalonic acid presented only the band corresponding to the geranyl-geranylated protein. In contrast, gemcitabine, with or without mevalonic acid 100 *μ*M, did not affect the geranyl-geranylation of p21*rhoA* on immunoblots (Figure 8A). In addition to this, fluvastatin determined the same concentration-dependent effects on p21*ras*, as shown by the appearance of a band shift representing the nonfarnesylated peptide (Figure 8B), as confirmed by image analysis (Figure 8B). Compared to controls, gemcitabine did not affect protein mobility in immunoblots due to inhibition of protein prenylation; on the contrary, the simultaneous treatment of fluvastatin with 100 *μ*M mevalonic acid markedly reduced the nonfarnesylated bands (Figure 8B). Furthermore, fluvastatin reduced the amount of the active, phosphorylated form of p42MAPK/ERK2 (Figure 8C), and the optical density ratio of phosphorylated/nonphosphorylated p42MAPK/ERK2 protein bands of treated cells appeared significantly decreased (*P*<0.05; Figure 8C). Mevalonic acid contrasted the concentration-dependent effects of fluvastatin, whereas gemcitabine did not affect the *ras* prenylation (Figure 8C).

### Immunocytochemical localisation of p21*rhoA* and p21*ras* proteins

**Figure 9:**
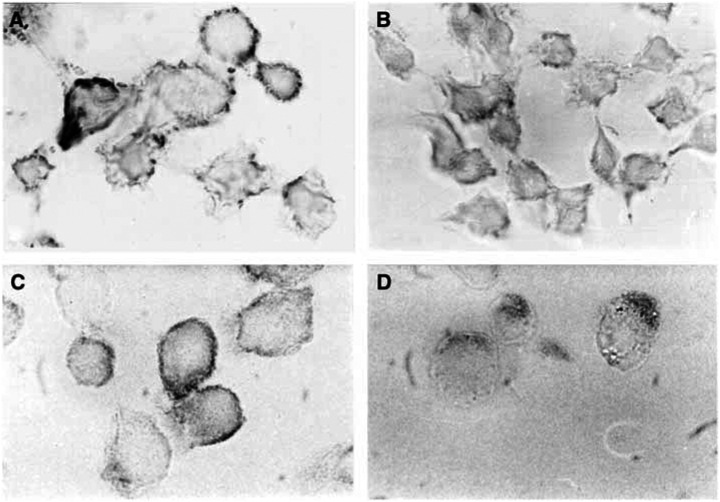
Immunohistochemical localisation (dark staining) of p21*rhoA* in MIAPaCa-2 cells control (**A**) and treated with fluvastatin (**B**), and p21*ras* in control cells (**C**) and treated with fluvastatin (**D**). Original magnification × 200.

Untreated MIAPaCa-2 cells showed a higher degree of immunoreactivity compared to fluvastatin-treated cells. A specific p21*rhoA* positivity was localised along the plasma membrane and the peripheral cytoplasmic boundaries of control cells (Figure 9A). In contrast, fluvastatin-treated cells displayed only a weak positivity homogeneously localised within the cytoplasm without specific immunostaining at the level of cell membranes (Figure 9B). Concerning anti-p21*ras*, the highest immunoreactivity was observed in control cells along the plasma membranes, with a faint immunostaining in their cytoplasm (Figure 9C). On the contrary, most of the fluvastatin-treated cells displayed a weak anti-p21*ras* immunoreaction localised mainly in the cytoplasm (Figure 9D); no specific localisation of the p21*ras* peptide was observed at the level of the plasma membranes or nuclei.

### Enhanced inhibition of tumour growth *in vivo* by the simultaneous combination of fluvastatin and gemcitabine

**Figure 10:**
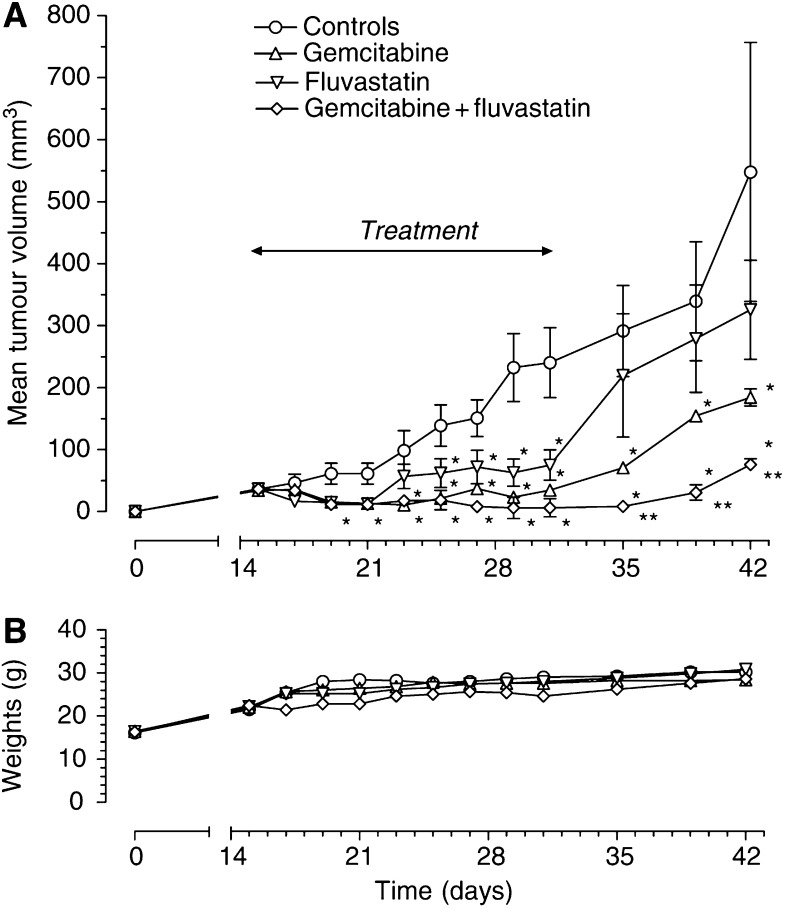
(**A**) Chemotherapeutic effect of gemcitabine 120 mg kg^−1^ i.p. four times at 3-day intervals and fluvastatin 30 mg kg^−1^ i.p. every 2 days alone or in combination on MIAPaCa-2 tumours xenotransplanted in CD nu/nu mice. ^*^P<0.05 with respect to controls; ^**^P<0.05 *vs* gemcitabine and fluvastatin alone. Symbols and bars, mean±s.e. (**B**) Body weight of MIAPaCa-2 tumour-bearing control mice and mice treated with gemcitabine and fluvastatin alone or in combination. No changes or decline in body weight were noted. Symbols and bars, mean±s.e.

MIAPaCa-2 cells injected s.c. in CD nu/nu mice grew quite rapidly and tumour masses became detectable 9 days after xenotransplantation. Tumours in control animals showed a progressive enlargement in their dimensions, and a mean volume of ∼550 mm^3^ was reached at the end of the experimental period (Figure 10A). Both fluvastatin and gemcitabine were able to inhibit tumour growth, although to different extents, and their therapeutic effect was significant starting on the 19th day after implant as compared to controls (Figure 10A). In the group of animals receiving the combined treatment with fluvastatin and gemcitabine, the reduction in tumour growth was significant already on day 19 with respect to controls (Figure 10A). The tumour growth curve of fluvastatin+gemcitabine showed a significant decrease during the 14-day schedule, divergent from that of controls, as well as from fluvastatin- and gemcitabine-treated animals, and at days 22 and 27 the tumour volume was significantly different from that of controls and of animals given fluvastatin and gemcitabine alone, respectively (Figure 10A). It is noteworthy that the combination of fluvastatin and gemcitabine resulted in an almost complete regression of tumour volumes (Figure 10A). Interestingly, all the drug schedules showed tumour relapses in all the treated groups at the end of the experiments, but with a significant delay in the case of the combination treatment. The toxicity profile was favourable and acceptable for both single and combination treatment, with no loss of weight throughout the course of the experiment (Figure 10B).

## DISCUSSION

The present study shows that fluvastatin inhibits proliferation, induces apoptosis in both human pancreatic cancer MIAPaCa-2 cells harbouring a p21*ras* mutation at codon 12 and in wild-type k-ras COLO320-DM cancer cells (although in different extent), and synergistically potentiates the cytotoxic effect of gemcitabine on these cancer cells. The pharmacologic effects of fluvastatin are prevented by treatment with mevalonic acid, suggesting that the demonstrated inhibition of geranyl-geranylation and farnesylation of cellular proteins, including p21*rhoA* and p21*ras*, plays a major role in its anticancer effect. Fluvastatin treatment also indirectly inhibits the phosphorylation of p42ERK2/MAPK, the cellular effector of *ras* and other signal transduction peptides. Indeed, a similar synergistic effect against cancer cell proliferation is obtained combining gemcitabine and PD98059, a MEK1/2 inhibitor that can block activation of downstream ERK-1/2 (Boucher *et al*, 2000), confirming the importance of this signal pathway in the observed experimental data. Moreover, fluvastatin treatments simultaneously increase the expression of the dCK, the enzyme required for the activation of gemcitabine, and reduce the 5′-NT, responsible for deactivation of gemcitabine, suggesting a possible additional role of these enzymes in the enhanced cytotoxic activity of gemcitabine in the combined treatment. Above all, the described *in vitro* effects were confirmed *in vivo*, with a significant enhancement of antitumour activity of the simultaneous administration of fluvastatin and gemcitabine.

Most studies of single-agent chemotherapy in patients with advanced adenocarcinoma of the pancreas showed low response rate and little impact on patient survival (Abbruzzese, 2002b). *Ras* oncogene is very frequently mutated in human pancreatic cancer, and its overexpression may be related to the neovascular and metastatic process (Rak and Kerbel, 2001). Therefore, the identification of new compounds able to affect *ras* and *ras*-related functions, in association with gemcitabine, one of the most active chemotherapeutic drugs used in pancreas neoplasm, may represent a rational approach to the therapy of advanced pancreatic cancer. Two of these agents, R-115777 and SCH-66336, are orally active heterocyclic compounds and already in phase II/III studies in patients (Dempke, 2003) with advanced pancreatic cancers to determine the extent of their clinical activity. Indeed, durable objective partial responses were noted in several patients (Dempke, 2003). Fluvastatin in our experiments increases the nonisoprenylated form of cellular proteins. Although farnesyl protein transferase inhibitors are currently being evaluated in phase II and III clinical trials (Zhu *et al*, 2003) and they were found to be well tolerated (Dempke, 2003), there are still no data on their long-term safety. On the other hand, statins have been in clinical use for more than 15 years with a well-known, yet favourable, toxicity profile, even if used for long periods. Fluvastatin has the same toxicity profile as other HMG-CoA reductase inhibitors, while it has not been associated with rhabdomyolysis and myopathy (Scripture and Pieper, 2001). Furthermore, fluvastatin has been safely administered at high doses in paediatric patients with cancer (Lopez-Aguilar *et al*, 1999). Indeed, micromolar concentrations of gemcitabine and fluvastatin have been reached in human plasma in pharmacokinetic studies (Kroep *et al*, 1999; Kantola *et al*, 2000), and these plasma levels are in the range of the antiproliferative activity found in the present study. Moreover, the administered fluvastatin dose in our *in vivo* experiment was based on the study by Ferrara *et al* (2003), who showed, for the same total dose given in 14 days, serum concentrations of fluvastatin in mice higher than 1.2 *μ*g ml^−1^, representing drug levels of ∼2 times the mean drug concentration in human plasma after a 40 mg oral dose and confirming the potential translation to the clinic of our data.

The chemotherapeutic activity of gemcitabine is well known (Bergman *et al*, 1996), and has been confirmed by MIA PaCa2 cells used in this study, whereas the antiproliferative activity of fluvastatin has been previously described only in breast cancer cells (Mueck *et al*, 2003). Treatment with fluvastatin is associated with tumour cell rounding, followed by cell detachment and apoptosis. This morphological change has also been previously described for the HMGCoA reductase inhibitor lovastatin *in vitro* on mesangial cells (Ghosh *et al*, 1997) and attributed to the inhibition of isoprenylation of small GTP-binding proteins that regulates the formation of actin stress fibres and focal adhesion plaques. In particular, in this study, fluvastatin inhibited the geranyl-geranylation of p21*rhoA,* a protein involved in cytoskeletal functions (Evers *et al*, 2000). Indeed, pancreatic cancer cells treated with fluvastatin accumulate *p21rhoA* in the cytoplasm, as shown by immunocytochemistry, because of the impairment of post-translational processing and lack of membrane association of the peptide.

To our knowledge, the data of the present study are the first findings to demonstrate that fluvastatin has an antiproliferative/proapoptotic effect on human pancreatic cancer cells. Fluvastatin may induce cells to become more susceptible to apoptosis by inhibiting the isoprenylation process, as demonstrated by the use of mevalonic acid as a pharmacological tool to reverse its antiproliferative and proapoptotic effects. In the combination treatment, fluvastatin increased apoptosis in a synergistic manner. By using the median-effect principle and the CI-isobologram technique, a synergism has been demonstrated between fluvastatin and gemcitabine *in vitro*, regardless of the schedule of administration, thus allowing for a dose reduction in order to obtain the same effect, with the consequence of lowering the toxicities of each drug in combination. Moreover, the *in vivo* simultaneous combination greatly inhibits human pancreatic tumours without any remarkable side effect on mice. At the present time, no association studies involving fluvastatin have been published yet; however, it has been previously shown that lovastatin enhanced the apoptosis induced by chemotherapeutic drugs, including 5-FU and cisplatin, in colon cancer cell lines (Agarwal *et al*, 1999). Furthermore, Holstein and Hohl (2001b) demonstrated synergism between paclitaxel and lovastatin on human cancer cell lines, whereas Feleszko *et al* (2002) showed an *in vivo* enhanced antitumour activity of doxorubicin in murine tumour models when associated with lovastatin.

In an effort to better explain the molecular basis of the demonstrated synergistic effects between gemcitabine and fluvastatin, the signal transduction pathway of the isoprenylated proteins and the activating metabolism of gemcitabine were further studied. The signal transduction to *ras* effectors was interrupted, as demonstrated by the lack of activation in fluvastatin-treated cells of p42MAPK/ERK2, a cytoplasmic serine/threonine proteine kinase, responsible for the transduction of mitogenic signals (Kolch, 2002). Indeed, the substitution of fluvastatin with the MAPK/ERK kinase inhibitor PD098059 in combination with gemcitabine produced similar synergistic results as the association of fluvastatin and gemcitabine. These data could suggest that MAPK activity suppression due to fluvastatin (through the isoprenylation inhibition) or PD098059 is a key step to enhance the cancer cell kill of gemcitabine, as also previously suggested (Nelson and Fry, 2001).

Moreover, in our experimental setting, the greater effect of the fluvastatin/gemcitabine combination could also be dependent on a higher concentration and prolonged half-life of the active mono-, di- and triphosphate metabolites of gemcitabine inside the pancreatic tumour cells due to the fluvastatin induction of the expression of dCK, which activates the drug by phosphorylation, and the reduction of 5′-NT, which removes the phosphate group from cytotoxic metabolites (Danesi *et al*, 2003).

In conclusion, the results of the present study demonstrate that the combination of gemcitabine and fluvastatin is an effective cytotoxic, proapoptotic treatment *in vitro* and *in vivo* against MIAPaCa-2 cells harbouring a mutated p21*ras* by a mechanism of action mediated, at least in part, by the inhibition of p21*ras* and *rhoA* prenylation. As stated recently by Wong *et al* (2002), ‘understanding the molecular mechanism of statin's anti-cancer action remains outstanding’ for the clinical management of patients and in this view, our data could contribute to a better understanding of the fluvastatin cytotoxic mechanism of action and to conceive new synergistic combinations in order to maximise the efficacy and minimise the drug-related toxicities *in vivo*, especially in the field of metastatic pancreatic cancer, where few effective therapeutic choices are presently available.

## Figures and Tables

**Table 1:** CI values for the three drug combinations at 75, 90 and 95% levels of inhibition of MIA PaCa-2 cell growth

	**CI values (mean±s.e.)**		
**Drug combination**	**75%**	**90%**	**95%**
Fluvastatin+gemcitabine	0.54±0.03	0.26±0.01	0.16±0.01
Fluvastatin → gemcitabine	0.66±0.04	0.33±0.02	0.22±0.01
Gemcitabine → fluvastatin	0.62±0.01	0.29±0.004	0.18±0.002

Arrows indicate the sequence of treatment.

**Table 2:** DRI values for the three combinations at 75, 90 and 95% levels of inhibition of MIA PaCa-2 cell growth

	**DRI values (mean±s.e.)**					
	**75%**		**90%**		**95%**	
**Drug combination**	**Fluvastatin**	**Gemcitabine**	**Fluvastatin**	**Gemcitabine**	**Fluvastatin**	**Gemcitabine**
Fluvastatin+gemcitabine	7.05±0.37	2.53±0.13	4.81±0.24	19.29±0.97	7.45±0.36	38.25±1.84
Fluvastatin → gemcitabine	5.78±0.36	2.08±0.13	3.47±0.22	14.98±0.88	5.58±0.31	28.63±1.60
Gemcitabine → fluvastatin	6.09±0.11	2.19±0.04	4.26±0.07	17.07±0.27	6.67±0.13	34.41±0.51

Arrows indicate the sequence of treatment.
